# Cytotoxic and anti-excitotoxic effects of selected plant and algal extracts using COMET and cell viability assays

**DOI:** 10.1038/s41598-021-88089-8

**Published:** 2021-04-19

**Authors:** Abeer Aldbass, Musarat Amina, Nawal M. Al Musayeib, Ramesa Shafi Bhat, Sara Al-Rashed, Najat Marraiki, Rania Fahmy, Afaf El-Ansary

**Affiliations:** 1 Biochemistry Department, College of Sciences, King Saud University, Riyadh, Saudi Arabia; 2 Department of Pharmacognosy, Pharmacy College, King Saud University, Riyadh, 11451 Saudi Arabia; 3 Botany and Microbiology Department, College of Sciences, King Saud University, Riyadh, Saudi Arabia; 4 Department of Ophthalmology, Faculty of Medicine, Cairo University, Giza, Egypt; 5 Department of Optometry, College of Applied Medical Sciences, King Saud University, Riyadh, Saudi Arabia; 6 Central Laboratory, Female Center for Scientific and Medical Studies, King Saud University, Riyadh, Saudi Arabia; 7 CONEM Saudi Autism Research Group, King Saud University, Riyadh, Saudi Arabia

**Keywords:** Drug discovery, Neuroscience

## Abstract

Excess glutamate in the central nervous system may be a major cause of neurodegenerative diseases with gradual loss and dysfunction of neurons. Primary or secondary metabolites from medicinal plants and algae show potential for treatment of glutamate-induced excitotoxicity. Three plant extracts were evaluated for impact on glutamate excitotoxicity-induced in primary cultures of retinal ganglion cells (RGC). These cells were treated separately in seven groups: control; *Plicosepalus. curviflorus* treated; *Saussurea lappa* treated; *Cladophora glomerate* treated. Cells were treated independently with 5, 10, 50, or 100 µg/ml of extracts of plant or alga material*,* respectively, for 2 h. Glutamate-treated cells (48 h with 5, 10, 50, or 100 µM glutamate); and *P. curviflorus*/glutamate; *S. lappa*/glutamate; *C. glomerata*/glutamate [pretreatment with extract for 2 h (50 and 100 µg/ml) before glutamate treatment with 100 µM for 48 h]. Comet and MTT assays were used to assess cell damage and cell viability. The number of viable cells fell significantly after glutamate exposure. Exposure to plant extracts caused no notable effect of viability. All tested plants extracts showed a protective effect against glutamate excitotoxicity-induced RGC death. Use of these extracts for neurological conditions related to excitotoxicity and oxidative stress might prove beneficial.

## Introduction

Neurodegeneration describes death of neurons in both central and peripheral nervous systems^1^. Neurodegenerative illness is characterized by progressive loss and dysfunction of neurons and neuron-supporting cells in the central nervous system (CNS). Herbal medicines and compounds extracted from plants, such as flavonoids, alkaloids, terpenes, celastrol, lycopene, and resveratrol, have attracted attention for their therapeutic potential^2^.

Neurological disorders are characterized by progressive nature, weak responses to treatment and a wide range of side effects caused by conventional therapeutic strategies encourage the search for complementary and alternative medicine. Plant extracts are traditionally used for the treatment of several neurological disorders^3^. Availability, cost efficiency and lower incidence of side effects of plant extracts offers significant advantages.

Medicinal plants exert beneficial effects in neurological disorders through multiple cellular and molecular mechanisms, including suppression of apoptosis, alleviation of inflammatory responses, and improvement of the antioxidant performance. Modulation of intracellular signaling is an essential role for preventive and therapeutic potential of plant extracts for neurological disorders, such as Alzheimer’s, Parkinson’s, Autism Spectrum Disorders, Multiple Sclerosis^1,4,5^.

Intracellular signaling that is repeatedly associated with neurological disorders, but is not given sufficient attention, is glutamate excitotoxicity. Overstimulation of glutamate receptors leading to neuronal damage. Exposure of neurons to excessive glutamate may cause deregulation of Ca2^+^ homeostasis, triggering oxidative stress, neuroinflammation, mitochondrial dysfunction and eventually neuron death. a consensus has developed that excitotoxicity is a common etiological mechanism in the pathogenesis of neurological and psychiatric disorders. Thus, targeting excitotoxic might be a useful therapeutic strategy^6^.

Phytochemicals are promising candidates for treating glutamate-induced excitotoxicity, and novel therapeutic approaches might arise from constituents from plant sources^7^. Various medicinal plants and natural products are used to treat neurodegenerative disorders^8,9^. Most recently, Afshari et al.^7^ reviewed the protective influences of some phytochemicals used to treat glutamate-induced neurotoxicity.

**Figure 1:**
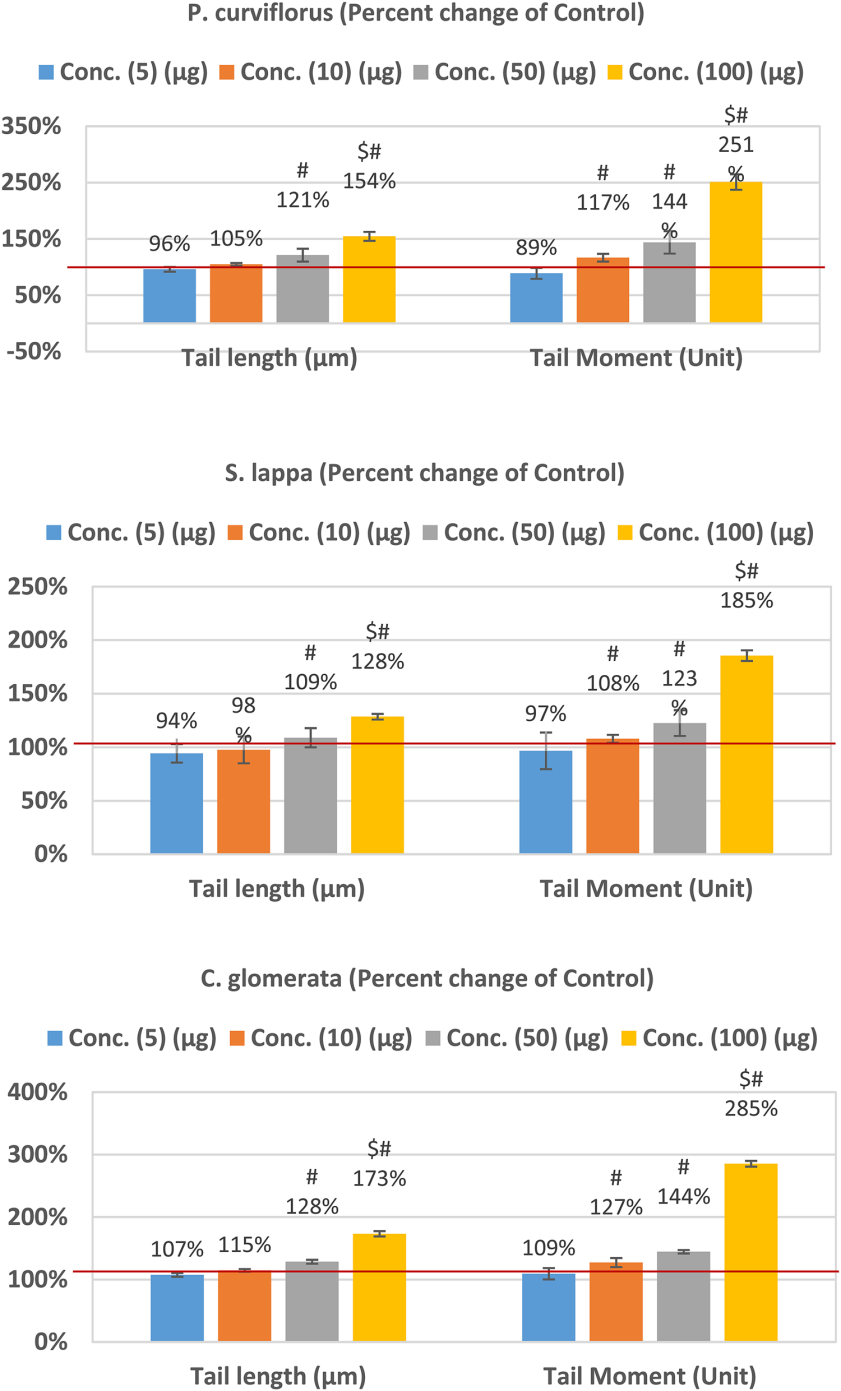
Percentage change of (**A**) *P. curviflorus* treated; (**B**) *S. lappa* treated; (**C**) *C. glomerate* tail length and tail moment compared to healthy untreated control RGCs.

*Plicosepalus curviflorus* (family Loranthaceae) is a medicinal plant grown in Saudi Arabia. Traditionally, stems were valued for cancer treatment in Yemen^10,11^. Various phytochemical studies of crude leaf extracts of *P. curviflorus* showed the presence of flavonoids, flavane gallates, sterols, and terpenoids^12,13^. Al-Taweel et al.^14^ and recently, Orfali, et al.^15^ isolate quercetin (P1), catechin (P2), and a flavane gallate–2S, 3R-3, 3′, 4′, 5,7-pentahydroxyflavane-5-Ogallate (P3) (Fig. 1) from aerial portions of *P. curviflorus.*

The herb, lappa (*Saussurea lappa*) in the family Asteraceae, is used in traditional ethnic medicine^16^. The antioxidant properties of this herb are attributed to the presence of the polyphenols and flavonoids^17^. These constituents bolster antioxidant defenses in a variety of pathophysiologic conditions characterized by oxidant/antioxidant imbalance^18^. Anti-inflammatory and antiapoptotic effects of *S. lappa* are reported^19,20^.

Special attention has been given to green macroalgae as sources of medicinal products^21^. *Cladophora glomerata* is a filamentous freshwater green alga, in the Ulvophyceae, a common family many aquatic ecosystems^22^. Numerous investigations of *Cladophora glomerata* show the presence of bioactive compounds that establish the species as a source of pharmaceutical and natural nutritional products^23–25^. Additionally, previous studies report that *C. glomerata* extracts exhibit properties to treat gastric ulcer, inflammation, pain, hypotension, and oxidative stress in different in vitro and in vivo experimental models^26^. Further, *C. glomerata,* enriched in chromium ions, promoted cell proliferation and, viability, and reduced apoptosis^27^.

Alterations in retinal function may imitate brain dysfunction in neurological and psychiatric disorders^28^ and may be useful in filling the need for novel approaches to indirectly examine brain function. The retina is a developmental and structural extension of the central nervous system (CNS). This work aims to address the role of glutamate excitotoxicity as a potential etiological mechanism in many neurological disorders and investigate the protective effects of *P. curviflorus,* S*. lappa,* and *C. glomerata* extracts on glutamate-induced neurotoxicity in retinal ganglion primary cell lines RGCs.

## Results

Phytochemical screening of methanolic extracts of *P. curviflorus* shoots, *S. lappa* roots and *C. glomerata* demonstrated the presence of anthraquinones, coumarins, tannins, flavonoids, terpenoids, alkaloids, cardiac glycosides, phlobatannins, and saponins. The extraction percentage yield of *P. curviflorus* shoots, *S. lappa* roots and *C. glomerata* varied from 2.55 to 9.55 with a descending order of *S. lappa* > *P. curviflorus* > *C. glomerata* (Table 1), indicating that the methanol extract of *S. Lappa* contains the highest concentration of extractable phytoconstituents. Table 1 summarizes that total phenolic components in extracts varied widely, ranging from 49.93 ± 1.8 to 122.82 ± 1.2 mg/g expressed as gallic acid equivalents (GAE). *S. lappa* extract showed the highest concentration of total phenolic contents followed by *P. curviflorus* extract. The flavonoid content is expressed as rutin equivalents, varied from 14.85 ± 0.5 to 39.52 ± 1.9 mg rutin equivalent/g extract (Table 1). The root extract of *S. lappa* exhibited the highest quantity of the highest amount of flavonoid contents.

**Table 1:** Total, free, bound phenolics content (mg GAE/g), flavonoid (mg RE/g) and extraction yield of methanol extract of *P. curviflorus*, *S. lappa* and *C. glomerata.*

Sample	Total phenolics	Free phenolics	Bound phenolics	Total flavonoids	Extraction yield (%)
*P. curviflorus*	89.36 ± 1.1	18.62 ± 0.9	54.92 ± 2.8	29.87 ± 0.8	8.53
*S. lappa*	122.82 ± 1.2	22.09 ± 0.6	104.34 ± 1.3	39.52 ± 1.9	9.52
*C. glomerata*	49.93 ± 1.8	12.04 ± 1.1	28.21 ± 1.5	14.85 ± 0.5	2.55

Each value in the table is expressed as mean ± S.D (n = 3).

**Figure 2:**
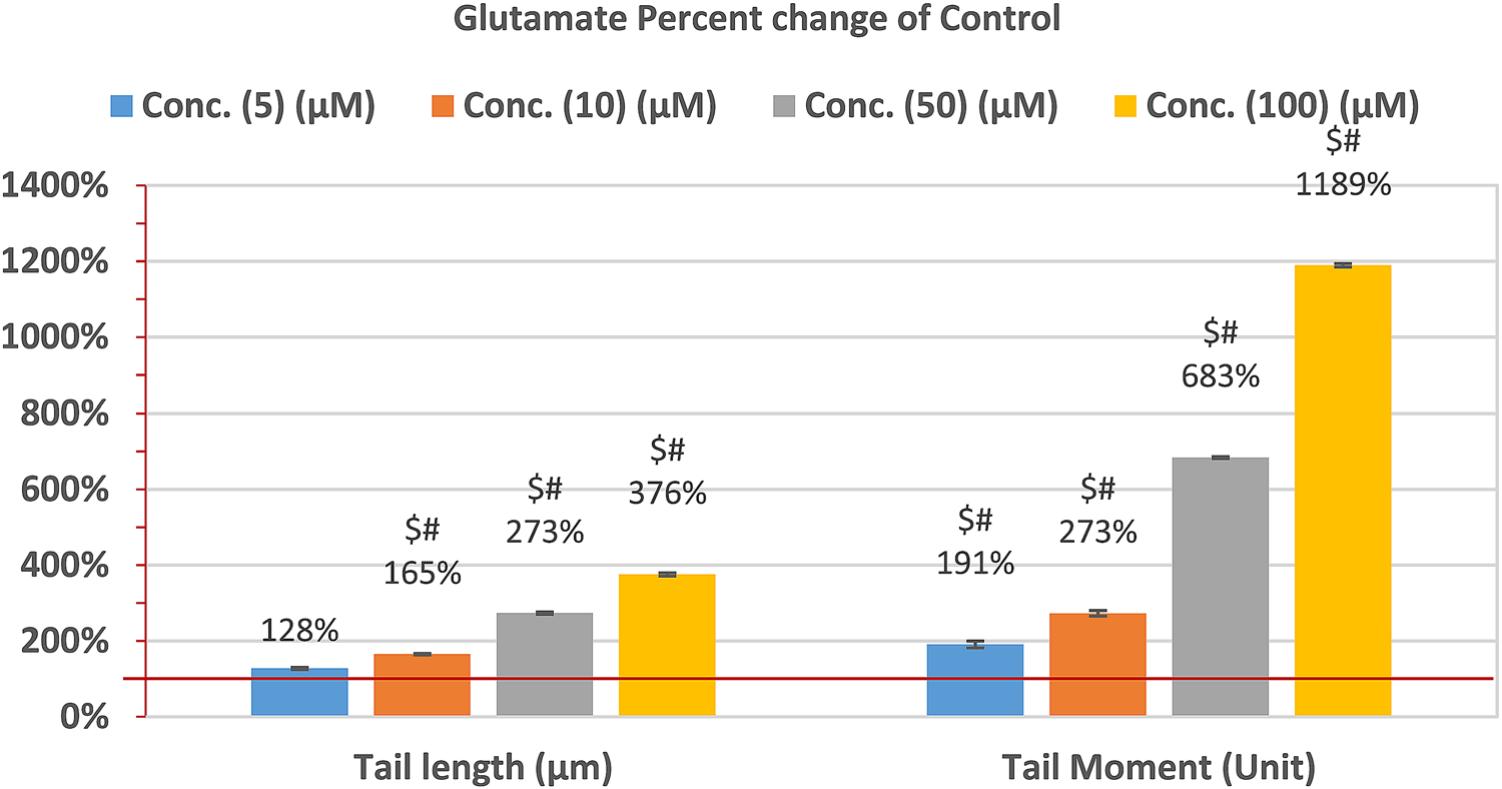
Percentage change of tail length and tail moment in glutamate-intoxicated RGCs compared to healthy untreated control cells.

The percentage of DNA migrating into the comet tail (indicating the presence of breaks) from the COMET assay is presented in Table 2 and Figs. 1 and 2. It can be easily noticed that the three plant extracts induced non-significant increase of tail length and tail moment at the highest concentration used (i.e. 100 µg) (Table 2). In contrast, glutamate induced dose dependent increase of both comet assay variables recording tail length values of 1.57 ± 0.13, 2.03 ± 0.2, 3.36 ± 0.32, and 4.62 ± 0.41 compared to a value of 1.23 ± 0.09 for control (Table 2). Much higher tail moments were also recorded in glutamate-intoxicated RGCs recording values of 2.88 ± 0.41, 4.12 ± 0.44, 10.32 ± 1.49, and 17.96 ± 4.09 compared to a tail moment value of 1.51 ± 0.10 in control cells (Table 2). Percentage changes of both comet variables in the three plants-treated and glutamate-treated cells are presented in Figs. 1 and 2 respectively compared to control-untreated RGCs.

**Table 2:** Comparison of control cells at various concentrations of glutamate and *P. curviflorus*; *S. lappa*; and *C. glomerate- treated.*

Parameters	Extracts	Concentration (µg plant extracts OR µM glutamate)			
		5	10	50	100
Tail length (µm)	Control	1.23 ± 0.09	1.23 ± 0.09	1.23 ± 0.09^#^	1.23 ± 0.09
	Glutamate	1.57 ± 0.13	2.03 ± 0.22	3.36 ± 0.32^$^	4.62 ± 0.41^$^
	*P. curviflorus*	1.18 ± 0.05	1.29 ± 0.03	1.49 ± 0.17^#^	1.90 ± 0.15^$#^
	*S. lappa*	1.16 ± 0.10	1.20 ± 0.15	1.34 ± 0.12^#^	1.58 ± 0.04^$#^
	*C. glomerata*	1.32 ± 0.04	1.41 ± 0.03	1.58 ± 0.05^#^	2.13 ± 0.09^$#^
Tail Moment (Unit)	Control	1.51 ± 0.10	1.51 ± 0.10^#^	1.51 ± 0.10^#^	1.51 ± 0.10
	Glutamate	2.88 ± 0.41	4.12 ± 0.44	10.32 ± 1.49	17.96 ± 4.09
	*P. curviflorus*	1.34 ± 0.13	1.76 ± 0.12^#^	2.17 ± 0.43^#^	3.79 ± 0.52^$#^
	*S. lappa*	1.46 ± 0.25	1.63 ± 0.06^#^	1.85 ± 0.22^#^	2.80 ± 0.14^$#^
	*C. glomerata*	1.65 ± 0.15	1.92 ± 0.14^#^	2.18 ± 0.06^#^	4.31 ± 0.20^$#^

Comparison among all cell treatments using One-Way ANOVA test with Multiple Comparisons (Dunnett test) to compare each group with the control group.

^$^p < 0.001, value between each cell group and control cells.

^#^p < 0.001 value between all group.

**Figure 3:**
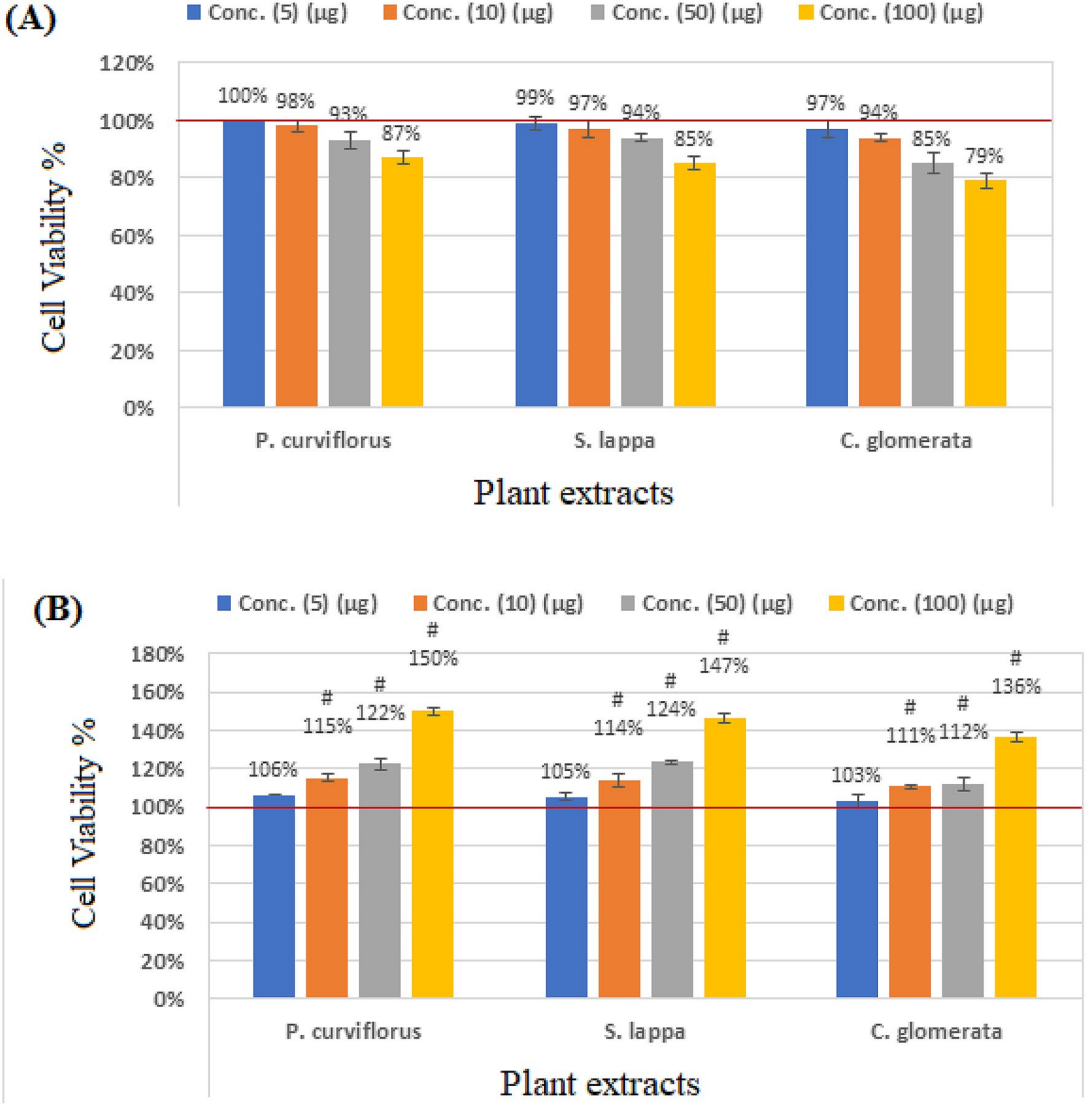
Percentage change of cell viability of RGCs treated with serial concentrations of *P. curviflorus* treated; *S. lappa* treated, and *C. glomerate* compared to control untreated-healthy cells (**A**) and glutamate-intoxicated cells (**B**).

The cytotoxic effects of plant extracts on RGCs proliferation after two-hour incubation was measured by MTT assay (Table 3 and Fig. 3A). Extracts caused a negligible but dose-dependent reduction in cell viability. In contrast, glutamate after 48 h of exposure to concentrations ranging from 5 to 100 µM, caused numbers of viable cells to fall significantly to 76% and 58% at 50 µM and 100 µM, respectively. Inhibition of cell proliferation was most pronounced at 100 µM concentration suggesting dose dependency. Cell viability of the plant extract treated RGCs cells are significantly different compared to glutamate intoxicated cells (Table 3 and Fig. 3B).

**Table 3:** Comparison with control cells (viability); glutamate-treated cells (viability).

Groups	Extracts	Plant extract concentrations			
		5 µg	10 µg	50 µg	100 µg
Control	Control	1.00 ± 0.00	1.00 ± 0.00	1.00 ± 0.00	1.00 ± 0.00
	*P. curviflorus*	1.00 ± 0.00	0.98 ± 0.02	0.93 ± 0.03	0.87 ± 0.02
	*S. lappa*	0.99 ± 0.02	0.97 ± 0.03	0.94 ± 0.01	0.85 ± 0.02
	*C. glomerata*	0.97 ± 0.03	0.94 ± 0.01	0.85 ± 0.03	0.79 ± 0.02

		Glutamate Concentrations			
		5 µM	10 µM	50 µM	100 µM
Glutamate	Glutamate	0.94 ± 0.02	0.85 ± 0.02^#^	0.76 ± 0.01^$#^	0.58 ± 0.03^$#^
	*P. curviflorus*	1.00 ± 0.00	0.98 ± 0.02^#^	0.93 ± 0.03^#^	0.87 ± 0.02^#^
	*S. lappa*	0.99 ± 0.02	0.97 ± 0.03^#^	0.94 ± 0.01^#^	0.85 ± 0.02^#^
	*C. glomerata*	0.97 ± 0.03	0.94 ± 0.01^#^	0.85 ± 0.03^#^	0.79 ± 0.02^#^

Comparison among all groups using Kruskal–Wallis test and using Mann–Whitney test to compare treated cells with controls (Non-parametric data).

Comparison among all groups using One-Way ANOVA test with Multiple Comparisons (Dunnett test) to compare treated cells with glutamate-induced cells (Parametric data).

^$^p < 0.001, value between each group and the control group.

^#^p < 0.001 value between all groups.

Cytoprotective effects were measured determined for cells pretreated with plant extracts (50 and 100 µg/ml) for 2 h followed by exposure to either 50 µM or 100 µM glutamate. Cell viability is expressed percent of control cells exposed to vehicle only. Control value was taken as 100%. Data are expressed as mean ± SD of three independent experiments (n = 3) (Table 4), significantly different at *p < 0.05, **p < 0.01). Extracts efficiently rescued cell viability of glutamate excitointoxicated RGCs and demonstrate much lower tail length as measure of DNA damage (Table 4). One hundred µg/ml aliquots of extracts of *P. curviflorus, S. lappa* or *C. glomerata* showed maximal protective effects of 27.58%, 31.03%, and 22.41%, respectively against excitotoxicity of a 100 µM glutamate (*p* < 0.01, n = 4).

**Table 4:** The protective effects of 50 µg and 100 µg of the three plant extracts in improving cell viability of 100 µM glutamate-treated cells.

Protective treatment	Extracts	Mean ± S.D
–	Control	1.00 ± 0.00
Extract (50 µg ) + Glutamate (100 µM)	Glutamate	0.58 ± 0.01^#^
	*P. curviflorus*	0.63 ± 0.02^$#^
	*S. lappa*	0.63 ± 0.02^$#^
	*C. glomerata*	0.65 ± 0.03^$#^
	Control	1.00 ± 0.00
Extract (100 µg ) + Glutamate (100 µM)	Glutamate	0.58 ± 0.03^#^
	*P. curviflorus*	0.74 ± 0.03^$#^
	*S. lappa*	0.76 ± 0.01^$#^
	*C. glomerata*	0.71 ± 0.01^$#^

Comparing between all groups using One-Way ANOVA test with Multiple Comparisons (Dunnett test) to compare each group with the glutamate group.

^$^p < 0.001, value between each group and the control group.

^#^p < 0.001 value between all groups.

Antioxidant activity is the ability of the antioxidants to protect the organism system towards the harmful effects of oxidative stress. In the current study, antioxidant capacity of methanol extract of *P. curviflorus, S. lappa,* and *C. glomerata* was assessed by DPPH and ABTS scavenging activity. The IC_50_ of DPPH and ABTS scavenging potentials of each extract was compared with ascorbic acid (Table 5).

**Table 5:** Antioxidant activity of *P. curviflorus*, *S. lappa*, and *C. glomerata* extracts.

Extracts	DPPH (IC_50_ μg/mL)	ABTS (IC_50_ μg/mL)
*P. curviflorus*	7.23 ± 0.05	60.26 ± 0.82
*S. lappa*	2.98 ± 0.68	35.15 ± 0.65
*C. glomerata*	45.32 ± 3.82	65.26 ± 1.22
Ascorbic acid	1.25 ± 0.01	24.23 ± 0.25

## Discussion

The fact that phenolic and flavonoid constituents are major active components in materials of natural product origin, including herbs, medicinal plants, algae, sponges and cyanobacteria is well documented. All the three tested samples showed noticeable variations in the content level of phenolic components studied among the species. The percentage of free phenolics varied considerably, highest was found in *S. lappa* (12.28%), followed by *P. curviflorus* (8.93%) and lowest in *C. glomerata* (4.99%). These free phenolics are considered as the responsible compounds for the cytotoxic, anti-excitotoxic and antioxidant properties under the experimental conditions applied in this study. The major types of phenolic components contributing in these activities mainly include simple phenolic compounds such as phenolic acids (hydroxybenzoic acids and hydroxycinnamic acids) and polyphenolic compounds including flavonoids, tannins, cumarins and curcuminoids.

Ethno-pharmacological research has provided probable finding about the use of plant origin new medications^7^. In traditional medicine, various therapeutic plants and natural products have been utilized to treat neurological disorders. Excitotoxicity is a well-accepted pathological mechanism of neurodegenerative disease^29^ caused by excess glutamate, a crucial excitatory neurotransmitter in mammals. Overstimulation of glutamate receptors leads to an overload of intracellular Ca^2+^, generation of free radicals and subsequent neuronal cell death^30^.

In the current study, single-cell gel electrophoresis (comet assay) was effective for evaluation of single-strand breaks of brain DNA post-exposure of RGCs to high levels of glutamate. The percentage of DNA migrating into the comet tail (indicating the presence of breaks) was not significantly elevated at 5 and 10 mM glutamate, but was increased from 1.23 ± 0.0941 (means ± SEM) in healthy untreated cells to 3.36 ± 0.32 and 4.62 ± 0.41 in the presence of 50 mM and 100 mM glutamate, respectively. Tail length and tail moment from the COMET assay are presented in Table 2 and Figs. 1 and 2 to describe DNA migration. Tail moment calculated by Olive et al.^31^ is principally useful in describing heterogeneity within a cell population since it identifies variations in DNA distributions within tails. Tail moment, as a derived measure, should be presented together with primary measurements (e.g., tail length and % tail DNA)^32,33^. In the present study, the other three measured Comet variables were submitted as “Supplementary data S1” (Tailed %, untailed % and % tail DNA).

Understanding the events of glutamate excitotoxicy and subsequent neuronal death is of critical importance in identifying novel therapeutic targets. NAMDA receptor overstimulation and other events are anticipated. Glutamate-induced elevated calcium levels over-activate several enzymes, including nitric oxide synthase, pro-apoptotic enzymes, phospholipases, protein kinases and phosphatases^34^. Some enzymes can also produce positive feedback loops to accelerate progression toward neuronal death through damage to cell membranes, cytoskeleton, and DNA^34–37^.

In the present study, Table 2 and Fig. 1, present results of measuring cytotoxic effects of plant extracts (*P. curviflorus; S. lappa,* and *C. glomerata)* using COMET assay in comparison to control healthy-untrated RGCs. Higher concentrations (100 µg/ml) of extracts exhibit slightly increased tail length and tail moment, but still much lower when compared to the excitotoxic effects of glutamate on RGCs. significant DNA damage was recorded in RGCs post-exposure to 50 and 100 µM glutamate (Table 2 and Fig. 2) presented as longer tail length, and greater tail moment are consistent with multiple previous studies. Collectively, non-significant cytotoxicity was observed in cultured rat cortical and hippocampal neurons exposed to 10–50 μM glutamate for 10 min, remarkable neuronal death was observed at higher concentrations of glutamate, 100 μM or greater^38,39^.

The cytotoxic effects of plant extracts on RGCs proliferation using MTT show a negligible but dose-dependent reduction in cell viability significantly different from glutamate-treated cells but, non-significantly different when compared to control healthy cells (Table 3 and Figs. 3A,B). This is going parallel with their effects on DNA using COMET assay.

The role of flavonoids as major components of the three phytochemical extracts is notable. Flavonoids can protect against glutamate excitotoxicity through inhibiting protein kinase activation as a signaling event. Flavonoids directly bind with multiple protein kinases such as Akt/PKB, Fyn, Janus kinase 1 (JAK1), mitogen-activated protein kinase kinase 1(MEK1), PI3K, mitogen-activated protein kinase kinase 4 (MKK4), Raf1, and chain-associated 70-kDa protein (ZAP-70) kinase. These kinases are specific proteins essential to intracellular signaling cascades associated with glutamate excitotoxicity^40,41^.

Phospholipase A2 is involved in many inflammatory reactions leading to disease progression and is a possible therapeutic target for attenuating glutamate excitotoxicity. Inhibition of this enzyme might address oxidative stress and neuroinflammation that contribute to disease^42,43^ Experimental results on PLA2-inhibition showed good inhibitory activity of quercetin as one of the major flavonoids in the three studied plant extracts^44^.

Differences in anti-apoptotic activity of individual flavonoids appear to reflect their configuration. Flavonoids display a scavenging activity against ROS with flavanols and flavonols showing the greatest potency. Flavanols (−) epigallocatechin gallate and quercetin at nontoxic doses of 50 µmol/L prevented H_2_O_2_-induced injury and sustained endothelial cell survival. Flavones, luteolin and apigenin, intensified H_2_O_2_-induced endothelial apoptosis, while epigallocatechin gallate and quercetin restored expression of antiapoptotic bcl-2 protein^45^. Activation of pro-apoptotic protein, caspase-3, is partially blocked by (−) epigallocatechin gallate and quercetin.

Significant ameliorative effects of plant extracts presented as lesser tail lengths and tail moments compared to 100 µM glutamate-induced DNA strand breaks (Table 4). This result could reflect the inhibitory action of flavonoids on protein kinases, phospholipases, and pro-apoptotic signaling. These major events are usually over-activated in response to exposure to high concentrations of glutamate. *S. lappa* shows the most potency followed by *P. curviflorus* and *C. glomerata.*

Possible flavonoid-related anti-excitotoxic effects of the three plant extracts for neurodegenerative disorders, such as Alzheimer’s and Parkinson’s diseases, is supported by multiple studies. Different feeding trials with the flavone, apigenin, show neuroprotective effects for memory and learning deficits, and reduction of fibrillar amyloid deposits in a rodent Alzheimer’s disease model. Additionally, restoration of cortical extracellular signal-regulated protein kinase 1 (ERK)/cAMP response element-binding protein (CREB)/BDNF pathway was observed. This pathway is involved in learning and memory deficits typically seen in Alzheimer’s disease patients^46,47^. Polyphenols are also as major components of P*. curviflorus* and *S. lappa* both of which significantly reduced the initial calcium peak in response to high concentrations of glutamate leading to protection from glutamate-induced cell death. Interestingly, the anti-excitotoxic effects reported in the present study can also find support in the study of Yang et al.^48^ which indicated an evidence that terpenes of plant origin protects against cerebral ischemic injury by inhibiting excitotoxicity through the modulation of the imbalance between excitatory glutamate against gamma-Aminobutyric acid (GABA) as inhibitory neurotransmitter, which may support the traditional use of terpenes for the treatment of stroke.

The DPPH radical scavenging activity of standard and methanol extracts tested (*P. curviflorus, S. lappa,* and *C. glomerata*) is summarized in Table 5. As shown in Table 5, all the three extracts demonstrated remarkable free radical scavenging activity with 84–95% DPPH radicals scavenged. The result obtained revealed that the methanol extract of *S. lappa* displayed the highest scavenging activity (lowest IC_50_ value; 2.98 ± 0.68 μg/mL) followed by *P. curviflorus* extract (IC_50_; 7.23 ± 0.05 μg/mL). However, *C. glomerata* extract showed moderate antioxidant activity (IC_50_; 45.32 ± 3.82). The excellent radical scavenging activity profile of *S. lappa* and *P. curviflorus* extract may be attributed to the presence of high content of hydrogen donating phenolic components in these extracts. Additionally, the hydroxyl group in the structural backbone of phenolics is the responsible contributors of antioxidant activity^49^.

The results of ABTS radical cation scavenging assay showed that the IC_50_ values of extracts were ranged 35.16–65.23 μg/mL (Table 5). Amongst the three tested extracts, *S. lappa* extract showed the highest activity with IC_50_ values of 35.15. *P. curviflorus* and *C. glomerata* extracts exhibited moderate activity with with IC_50_ values of 60.26 μg/mL and 65.26 μg/mL, respectively. Ascorbic acid was used as standard with IC_50_ values 24.23 μg/mL. The outcome the study was analyzed that the higher the concentration of phenolic components in the extracts, higher antioxidant capacity values they possess, which is clearly in agreement with the results observed in previous studies^50^. In conclusion, under the conditions employed in the present work, *P. curviflorus; S. lappa,* and *C. glomerata* extracts presented chemoprotection against the cytotoxic effects of glutamate on RGC.

## Material and methods

### Chemicals

Methanol (MeOH, 99.8%), dimethyl sulfoxide (DMSO, ≥ 95%), acetic anhydride (≥ 99%), sulfuric acid (H_2_SO_4_, 99.9%), papain, l-cysteine (97%), bovine serum albumin (BSA), ovomucoid, Dulbecco’s Phasphate buffer saline (Dulbecco’s PBS), Poly-d-lysine (PDL), 3,5,3-triiodo-l-thyronine (T3, ≥ 95%), l-thyroxine (T4, ≥ 98%), sodium pyruvate (≥ 99%), N-acetyl-l-cysteine (NAC), 2,2-diphenyl-1-picrylhydrazyn (DPPH), penicillin–streptomycin, glutamate, Caged hydrate (C_16_H_19_N_3_O_7_ × ·H_2_O), sodium hydroxide (NaOH), ethylenediaminetetraacetic acid disodium salt (ETDA-Na_2_), ethidium bromide (Et/br, ≥ 95%) 2,2′–azino-bis(3-ethylbenzthiazoline-6-sulfonic acid) (ABTS), were acquired from Sigma-Aldrich (Hamburg, Germany).

### Plant material

The shoots of *P. curviflorus* and roots of *S. lappa* were collected from Abha, Asir region of Saudi Arabia in March 2013 and Dhara peak, Srinagar, Jammu and Kasmir region of India in June 2016, respectively. However, the freshwater macroalgae *C. glomerata* biomass were collected by hand from the side walls of red sea of Jeddah (21° 42′ N 39° 10′ E), Saudi Arabia in September 2018. No specific permission was required for this location. Plant species as well as algal biomass were identified and authenticated by Dr Mohamed Yousef, a taxonomist in the Pharmacognosy Department, College of Pharmacy, King Saud University, Riyadh, Saudi Arabia. A voucher specimen with catalog Nos: (PC-3-2143), (SC-7806) and (CG-5018) were submitted to the herbarium of the same department for *P. curviflorus S. lappa*, and *C. glomerata* respectively. All the three samples were air-dried, coarse powdered, and preserved in airtight bags until extracted in the laboratory. This study complies with the local national regulations for the use of cultivated plants in experimental studies.

### Extraction of plant samples

Methanol was selected as an organic extractant using the ratio of 1:4 plant material. One thousand grams of powdered *P. curviflorus* shoots, *S. lappa* roots and *C. glomerata* biomass were individually soaked in 3 L of methanol in an airtight glass containers for four days at ambient temperature with shaking at 2 h intervals for maximum extraction of bioactive constituents^51–53^. Liquid extracts were separated from solid residues by filtration through Whatman No. 1 filter paper. All the experiments were performed in two replications under similar conditions. The organic solvent of each combined extract was evaporated to dryness in a rotaevaporator under reduced pressure at a temperature of 40 °C to yield dark brown (85.3 g), brown (92.5 g) and dark green (25.5 g) residues for *P. curviflorus* shoots, *S. lappa* roots and *C. glomerata* biomass, respectively. The dried residues were transferred to tightly stoppered glass tubes and stored at 5 °C until further use.

### Phytochemical screening

A qualitative standard screening method was adopted to determine major classes of phytoconstituents present in methanol extracts. Chemical tests used qualitative phytochemical screening included: anthraquinones (Borntrager’s test), phenolic compounds (Shinoda test), flavonoids (Ferric chloride test), steroids–triterpenoids (Libermann–Burchard test), tannins (lead acetate test), quinones (Borntraguer test), alkaloids (Dragendorff’s test), saponins (Rosenthaler test), cumarines (KOH reaction), iridoids (Trim–Hill test), lignins (Labat test), resins (acetic anhydride-sulfuric acid test), and cardiac glycosides (Keller–Killiani test)^54^.

#### Preparation of retinal cell suspensions

Newborn Sprague-Dawley rats were used for retinal cell preparation. Cells were isolated on postnatal days 1–4 and incubated in cold calcium and magnesium-free Earle’s Balanced Salt Solution and Hank’s Balanced Salt Solution containing 5% papain, 0.24% l-cysteine, and 10 U/ml DNase I for 30 min. Ovomucoid solution containing 0.1% bovine serum albumin, 0.1% ovomucoid and 1% DNase I was then used to stop papain activity. Cells were centrifuged at 200 × *g* for 10 min and suspended in minimal essential medium (MEM) amino acid solution containing 0.5 mg/ml bovine serum albumin (BSA). Finally, cells were filtered through a 40 μm mesh to obtain single-cell suspensions. The protocol of this work was approved by and carried out in accordance to the guidelines of College of science ethical committee, King Saud University No.: 4/67/352670. Our study was carried out in compliance with the ARRIVE guidelines.

#### RGC purification

##### Preparation of panning dishes and cell culture dishes/plates

Rabbit anti-rat macrophage/Thy-1 antibody-coated Petri dishes were used for negative and positive selection of cells. Panning plates were incubated overnight at 4 °C and rinsed three times with Dulbecco’s PBS (1×; 0.9 mM CaCl_2_, 0.49 mM MgCl_2_-6H_2_O, 137.9 mM NaCl, 2.67 mM KCl, 8.06 mM Na_2_HPO_4_-7H_2_O, 1.47 mM KH_2_PO_4_, pH 7.4; D-PBS; Gibco) before use. 1 × Poly-d-lysine stock (PDL; Sigma-Aldrich) was added to culture plates and incubated overnight at room temperature. Mouse laminin was added to dried cell culture plates and incubated at 37 °C for 2 h. The plates were rinsed with D-PBS three times before use.

#### Cell culture

Desired density of purified RGCs was seeded on PDL- and laminin-coated coverslips in prewarmed RGC growth medium at 37 °C in 5% CO_2_ incubator. RGC growth medium contained Neurobasal medium, BSA (0.1 mg/ml), transferrin (0.1 mg/ml), progesterone (60 ng/ml), putrescine (16 µg/ml), selenium (40 ng/ml), 3,5,3-triiodothyronine T3 (40 ng/ml), thyroxine T4 (40 ng/ml), B27 (20 µl/ml), sodium pyruvate (1 mM glutamine (2 mM), N-acetyl-l-cysteine (NAC, 5 µg/ml), insulin (5 µg/ml), forskolin (5 µM), brain-derived neurotrophic factor (BDNF, 50 ng/ml), ciliary neurotrophic factor (CNTF, 10 ng/ml), basic fibroblast growth factor (bFGF, 10 ng/ml), and penicillin–streptomycin (100 U/ml). Fifty percent of medium was replaced after 3 days.

### Cell treatment

Pure RGCs were divided into seven treatment groups: control (untreated); plant extract treated independently with 5, 10, 50, or 100 µg/ml of *P. curviflorus, S. lappa* or *C. glomerata*. Control and treated cells were exposed to extracts for 2 h. Glutamate-treated cells were exposed to glutamate for 48 h using 5, 10, 50, or 100 µM glutamate). Finally, cells pretreated individually with extracts (50, or 100 µg/ml) were exposed to 100 µM glutamate for 48 h.

#### Comet assay

The method described by Singh et al.^55^ was used for the comet assay. Cells were treated with test material for 24 h in Petri dishes. Cells were trypsinized (0.1% for 4 min), suspended, and centrifuged for 10 min at 800 rpm. Next, 600 μl of 0.8% low-melting agarose was added to the cell suspension and transferred to pre-coated agarose slides. The coated slides were immersed in lysis buffer (0.045 M TBE, pH 8.4, containing 2.5% SDS) for 20 min. The slides were placed on a gel electrophoresis and covered with ice-cold alkaline solution (300 mM NaOH and 1 mM Na2 EDTA, pH 13) in the dark at 0 °C for 20 min, before the electrophoretic run. The electrophoresis conditions were 2 V/cm for 20 min and 100 milliampere (mA). Ethidium bromide (20 μg/ml at 4 °C) was used for staining. DNA fragment migration patterns of 100 cells for each dose level were evaluated with a fluorescence microscope. DNA damage was measured as tail length (TL = distance of DNA migration from the center of the body of the nuclear core) and tail intensity of DNA (TI = % of genomic DNA that migrated during the electrophoresis from the nuclear core to the tail).

#### Determination of cell viability

MTT test was used to measure cell viability using RGC seeding density of 0.625 × 10^5^ (i.e. 62,500 cells/ml) in a 100 µL media. To assess viability, the medium was exchanged for an MTT working solution (5 mg/ml in cell culture medium) and incubated for 4 h at 37 °C. Afterwards, the reaction was terminated by adding 200 µL of DMSO to each well for 15 min. The absorbance was recorded at 490 nm using a micro plate reader. The results are presented as a percentage of control (untreated cells) or glutamate excitotoxicity.

#### DPPH radical scavenging assay

The antioxidant potential of plant extracts was evaluated by UV specterophotometrically against against 2,2-diphenyl-1-picrylhydrazyl (DPPH) and 2,2-Azino-Bis-(3-ethylbenzothiazoline-6-Sulfonic Acid) (ABTS) radical.

DPPH free radical scavenging activity of each plant extract was performed according to previously described method by Huang et al.^56^, with slight modification. Briefly, 2.5 ml of each sample extract was thoroughly mixed with 0.5 ml of freshly prepared 0.2 ml DPPH in ethanol solution and allowed to stand for 30 min at room temperature. The blank samples were prepared by mixing the same amount of ethanol and DPPH. After that, each reaction mixture was examined for DPPH radical scavenging effect by measuring the absorbance at 517 nm against blank samples on a UV–Vis spectrophotometer. Lower the value of absorbance of reaction mixture higher would be the free radical scavenging activity. The concentration of extract causing the 50% inhibition (IC_50_) was determined from the graph plot of percentage inhibition versus extract concentration. Ascorbic acid was used as a standard. Following equation was used to calculate the radical scavenging activity:

$${\text{DPPH radical scavenging }}\left( \% \right) = \frac{Ab - Aa}{{Ab}} \times 100$$

where *Ab* and *Aa* is the absorption of the blank sample and the extract, respectively.

#### ABTS radical scavenging assay

ABTS cation scavenging activity of plant extracts was conducted by obeying a procedure described in previous study^57^. Briefly, ABTS reagent was prepared by mixing equal volumes of freshly prepared stock solutions of ABTS (7 mM) and potassium persulphate (2.4 mM) and were allowed to react in the dark for 12 h at ambient temperature. After 12 h incubation, the resultant dark colored ABTS reagent solution was diluted with ethanol until an absorbance of 0.700 ± 0.005 at 734 nm was attained. 200 μl of each test sample solution was treated with 2 mL of ABTS stock solution. The reaction mixture was vortoxed for 30 min and absorbance was recorded at 734 nm. Similarly, different concentrations (1–100 μg/mL) of ascorbic acid was treated with ABTS solution. The amount of extract required to decrease the absorbance of ABTS by 50% (IC_50_) was determined graphically. Following equation was applied to calculate the antioxidant activity of each extract:

$${\text{ABTS radical scavenging }}\left( \% \right) = \frac{Ab - Aa}{{Ab}} \times 100$$

### Statistical analysis

Data were analyzed using the Statistical Package for the Social Sciences (SPSS, Chicago, IL, USA). Results are presented as mean ± standard error (SEM). All statistical comparisons among the control, *P. curviflorus* treated; *S. lappa* treated; *C. glomerate* -treated, and glutamate-treated groups used one-way analysis of variance (ANOVA) complemented with Dunnett’s test for Multiple Comparisons. Significance was considered *p* < 0.05.

## Supplementary Information

Supplementary Information.
