# Supplementary material for: Cytotoxic and anti-excitotoxic effects of selected plant and algal extracts using COMET and cell viability assays
Source: Sci Rep. 2021 Apr 19;11:8512. doi: 10.1038/s41598-021-88089-8 (PMC8055880; doi:10.1038/s41598-021-88089-8)
Supplement: Supplementary file 1 — Supplementary Information. [file 41598_2021_88089_MOESM1_ESM.docx]

**Table S1: Tailed %, un-tailed %, and Tail DNA (%)** of ***P. curviflorus*; *S. lappa*; and *C. glomerate- treated*** **and glutamate-treated compared to healthy untreated RGCs.**

| Parameters | Extracts | Concentration | | | | | | | |
| --- | --- | --- | --- | --- | --- | --- | --- | --- | --- |
|  |  | 5 | | 10 | | 50 | | | 100 |
| Tailed *(%)* | *Control* | | 3.00 ± 1.00 | | 3.00 ± 1.00 | | 3.00 ± 1.00 | 3.00 ± 1.00 | |
|  | *Glutamate* | | 5.33 ± 1.15 | | 8.67 ± 0.58 | | 20.00 ± 1.73 | 27.67 ± 2.52 | |
|  | *P. curviflorus* | | 3.67 ± 0.58 | | 4.00 ± 1.00 | | 4.67 ± 0.58 | 5.67 ± 1.53 | |
|  | S. lappa | | 1.67 ± 0.58 | | 2.67 ± 0.58 | | 3.67 ± 0.58 | 5.33 ± 0.58 | |
|  | *C. glomerata* | | 4.00 ± 1.00 | | 4.00 ± 1.00 | | 3.33 ± 0.58 | 7.67 ± 1.15^$^ | |
| Untailed (%) | Control | | 97.00 ± 1.00 | | 97.00 ± 1.00 | | 97.00 ± 1.00 | 97.00 ± 1.00 | |
|  | Glutamate | | 94.67 ± 1.15 | | 91.33 ± 0.58 | | 80.00 ± 1.73 | 72.33 ± 2.52 | |
|  | *P. curviflorus* | | 96.33 ± 0.58 | | 96.00 ± 1.00 | | 95.33 ± 0.58 | 94.33 ± 1.53 | |
|  | *S. lappa* | | 98.33 ± 0.58 | | 97.33 ± 0.58 | | 96.33 ± 0.58 | 94.67 ± 0.58 | |
|  | *C. glomerata* | | 96.00 ± 1.00 | | 96.00 ± 1.00 | | 96.67 ± 0.58 | 92.33 ± 1.15^$^ | |
| Tail DNA (%) | Control | | 1.24 ± 0.16 | | 1.24 ± 0.16 | | 1.24 ± 0.16 | 1.24 ± 0.16 | |
|  | Glutamate | | 1.57 ± 0.13 | | 2.03 ± 0.22 | | 3.36 ± 0.32 | 4.62 ± 0.41 | |
|  | *P. curviflorus* | | 1.14 ± 0.10 | | 1.36 ± 0.06 | | 1.45 ± 0.12 | 1.99 ± 0.13^$#^ | |
|  | *S. lappa* | | 1.25 ± 0.11 | | 1.36 ± 0.13 | | 1.38 ± 0.05 | 1.77 ± 0.04^$#^ | |
|  | *C. glomerata* | | 1.25 ± 0.11 | | 1.36 ± 0.13 | | 1.38 ± 0.05 | 2.02 ± 0.06^$#^ | |

^$^; p < 0.001, value between each cell group and control cells

^#^; p < 0.001 value between all group

Comparison among all cell treatments using One-Way ANOVA test with Multiple Comparisons (Dunnett test) to compare each group with the control group.


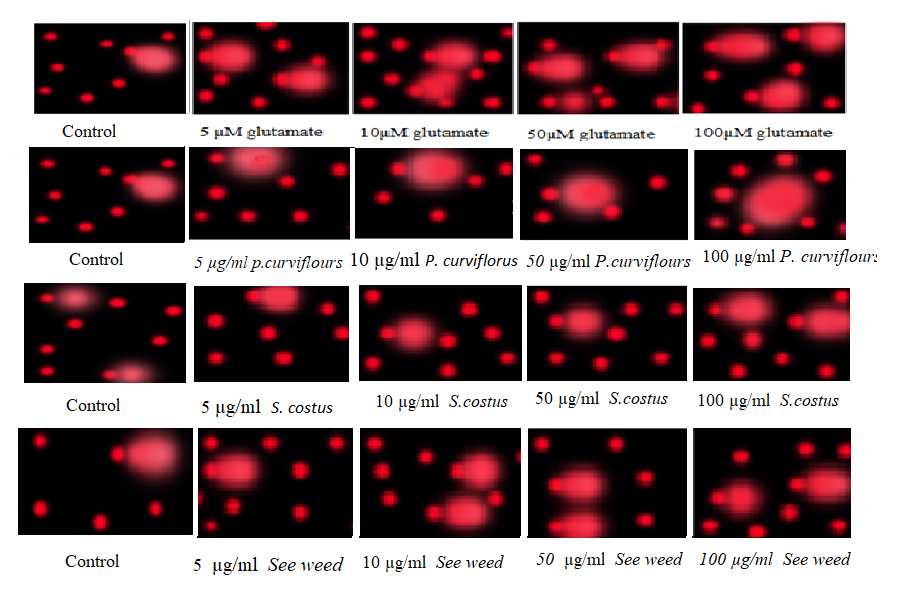


**Figure S1: Measurements of glutamate-induced DNA damage by comet assay in control, glutamate excitotoxic, and - *P.curviflorus* treated ; *S.lappa* treated; *C.***


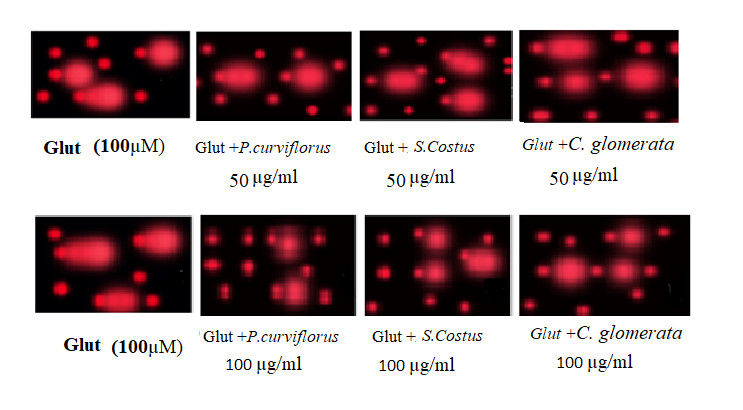


**Figure S2: Protective effects of *P. curviflorus,* S*. lappa,* and *C. glomerata* against glutamate-induced DNA damage measured by COMET assay *glomerate* treated RGCs**
